# Benign adrenal adenomas secreting excess mineralocorticoids and glucocorticoids

**DOI:** 10.1530/EDM-13-0042

**Published:** 2013-09-23

**Authors:** Vivienne Yoon, Aliya Heyliger, Takashi Maekawa, Hironobu Sasano, Kelley Carrick, Stacey Woodruff, Jennifer Rabaglia, Richard J Auchus, Hans K Ghayee

**Affiliations:** Department of Internal Medicine/Division of Endocrinology and MetabolismVA North Texas Health Care System and the University of Texas Southwestern Medical Center5323 Harry Hines Boulevard, Dallas, Texas, 75390-8857USA; 1 Department of PathologyTohoku University School of Medicine and Hospital at SendaiSendaiJapan; 2 Department of PathologyUniversity of Texas Southwestern Medical CenterDallas, TexasUSA; 3 Department of SurgeryUniversity of Texas Southwestern Medical CenterDallas, TexasUSA; 4 Department of Internal Medicine/Division of Metabolism Endocrinology and DiabetesUniversity of Michigan Health SystemAnn Arbor, MichiganUSA

## Abstract

Objective: To recognize that benign adrenal adenomas can co-secrete excess aldosterone and cortisol, which can change clinical management.

Methods: We reviewed the clinical and histological features of an adrenal tumor co-secreting aldosterone and cortisol in a patient. Biochemical testing as well as postoperative immunohistochemistry was carried out on tissue samples for assessing enzymes involved in steroidogenesis.

Results: A patient presented with hypertension, hypokalemia, and symptoms related to hypercortisolism. The case demonstrated suppressed renin concentrations with an elevated aldosterone:renin ratio, abnormal dexamethasone suppression test results, and elevated midnight salivary cortisol concentrations. The patient had a right adrenal nodule with autonomous cortisol production and interval growth. Right adrenalectomy was carried out. Postoperatively, the patient tolerated the surgery, but he was placed on a short course of steroid replacement given a subnormal postoperative serum cortisol concentration. Long-term follow-up of the patient showed that his blood pressure and glucose levels had improved. Histopathology slides showed positive staining for 3β-hydroxysteroid dehydrogenase, 11β-hydroxylase, and 21 hydroxylase.

Conclusion: In addition to the clinical manifestations and laboratory values, the presence of these enzymes in this type of tumor provides support that the tumor in this patient was able to produce mineralocorticoids and glucocorticoids. The recognition of patients with a tumor that is co-secreting aldosterone and cortisol can affect decisions to treat with glucocorticoids perioperatively to avoid adrenal crisis.

**Learning points:**

- Recognition of the presence of adrenal adenomas co-secreting mineralocorticoids and glucocorticoids.
- Consideration for perioperative and postoperative glucocorticoid use in the treatment of co-secreting adrenal adenomas.

## Background

The rise in the use of cross-sectional imaging has led to the incidental discovery of adrenal adenomas in up to 4% of the middle-aged population and 10% of the elderly population (1). Adrenal adenomas can be functional or nonfunctional, with 85% of them being nonfunctional. The 15% of adenomas that are functional are typically categorized as single-hormone-secreting adrenal adenomas (2). A prospective study evaluating the diagnostic utility of postural stimulation testing, computed tomography (CT), and adrenal vein sampling (AVS) in the assessment of primary aldosteronism, however, has reported a 14% incidence of adrenal adenomas co-secreting aldosterone and cortisol (3).

With excess mineralocorticoids and glucocorticoids, patients can develop uncontrolled hypertension and hypokalemia. The recognition of co-secreting adrenal adenomas will have implications for management in terms of perioperative use of glucocorticoids to avert the possibility of adrenal crisis. We present a case of an adrenal adenoma secreting excess mineralocorticoids and glucocorticoids and also discuss how this case was managed.

## Case presentation

A 52-year-old man presented with a history of uncontrolled hypertension, hypokalemia, and type 2 diabetes since the age of 36 years. He had a history of a right adrenal nodule found 2 years previously during a workup for hypertension and hypokalemia. The patient did not have any complaints of skin thinning or easy bruising. He was taking four medications for his blood pressure including lisinopril 40 mg twice daily, amlodipine 10 mg daily, metoprolol 50 mg twice daily, hydrochlorothiazide 25 mg daily, and potassium 40 mEq daily.

## Investigation

**Figure 1:**
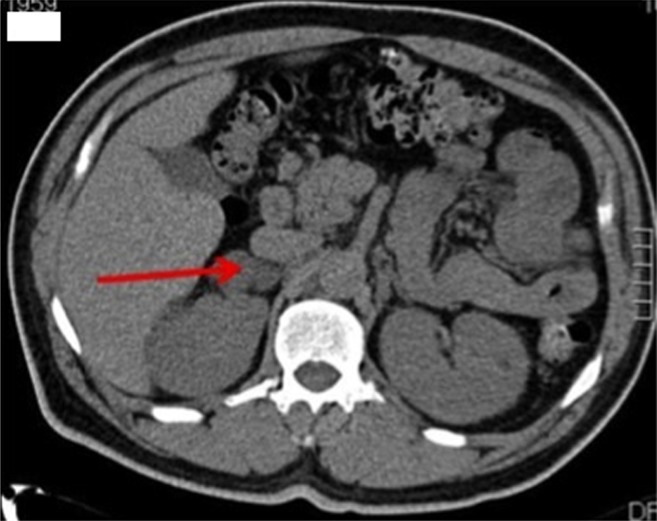
Preoperative CT scan showing right adrenal adenoma in the presented case.

During a workup for hypertension and hypokalemia (Table 1), serum aldosterone concentration of the patient was found to be 11 ng/dl with a plasma renin activity (PRA) of 0.25 ng/ml per h, with an aldosterone:renin ratio of 44 while taking lisinopril. Early-morning plasma adrenocorticotropic hormone (ACTH) concentration was 16 pg/ml with a serum cortisol concentration of 16.1 μg/dl and a suppressed DHEA-S concentration; urinary free cortisol concentration was normal. The patient underwent a 1-mg overnight dexamethasone suppression test during which serum cortisol concentration decreased to 3.9 μg/dl, which is greater than the generous 1.8 μg/dl cutoff to rule out Cushing's syndrome based on the current Endocrine Society guidelines (4). Midnight salivary cortisol concentrations were abnormal at 0.110, 0.140, and 0.220 μg/dl (reference value <0.090 μg/dl). As per the Endocrine Society guidelines, pheochromocytoma was also ruled out with normal 24-h urinary catecholamine and metanephrine levels. An abdominal CT scan showed growth of the right adrenal nodule from 3.5 to 4.6 cm in its greatest dimension over a 2-year period (Fig. 1). The right adrenal mass was homogeneous with a density of 18 Hounsfield units. Since his blood pressure was difficult to control, spironolactone was later used with marked improvement.

**Table 1:** Preoperative and postoperative biochemical evaluation in the presented case

**Laboratory values**	**Patient**		
	Preoperative	Postoperative day 1	Postoperative 6 weeks
Sodium (133–145 mmol/l)	140		139
Potassium (3.3–5.1 mmol/l)	3.6		4.5
Renin (0.25–5.82 ng/ml per h)	0.25		0.5
Aldosterone (<32 ng/dl)	11		6
ACTH (6–50 pg/ml)	16		35
Cortisol (6.7–22.6 μg/dl)	16.1	1.0	10.9
Cortisol after low-dose DST test (normal suppression <1.8 μg/dl)	3.9		
DHEA-S (80–560 μg/dl)	<30		
24-h urinary free cortisol (4–50 μg/24 h)	3.8		
Midnight salivary cortisol (reference value <0.090 μg/dl)	Salivary #1: 0.110		
	Salivary #2: 0.140		
	Salivary #3: 0.220		

## Treatment

**Figure 2:**
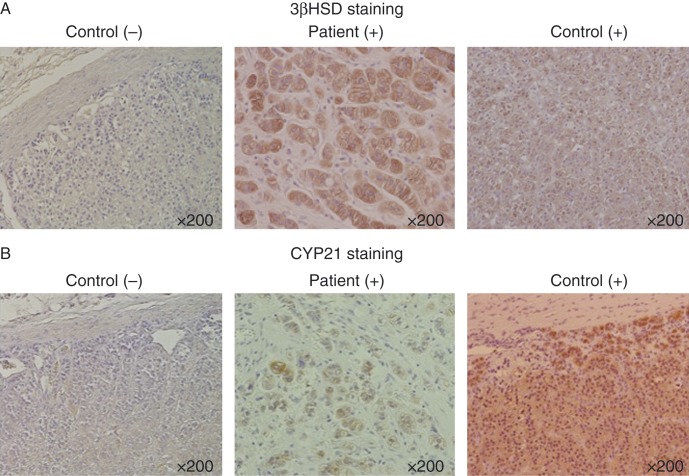
Histopathologic immunostaining of enzymes 3βHSD (A) and CYP21A2 (B) in the presented case. (−), negative staining; (+), positive staining.

In most circumstances, isolated primary aldosteronism is confirmed with a salt suppression test, followed by AVS for the purpose of lateralization in preparation for surgery. In our case, because we were concerned about adrenocortical carcinoma due to enlargement, >4 cm adrenal mass with evidence of autonomous cortisol production, a right adrenalectomy was carried out without further studies. An adrenal adenoma without evidence of malignancy was confirmed histopathologically. Immunohistochemistry of the tumor tissue was carried out for steroidogenic enzyme expression. The samples stained positive for 3β-hydroxysteroid dehydrogenase (3βHSD; Fig. 2A) and 11β-hydroxylase (CYP11B1) (not shown) and for 21 hydroxylase (CYP21A2) (Fig. 2B).

## Outcome and follow-up

The patient was found to have a cortisol concentration of 1.0 μg/dl the first morning postoperatively. Due to concern regarding an adenoma co-secreting cortisol, the patient was given 10 mg of hydrocortisone a day. Six weeks after adrenalectomy, repeat testing showed the resolution of primary aldosteronism with a morning aldosterone concentration of 6 ng/dl and PRA of 0.5 ng/ml per h. During this period, the patient held his hydrocortisone a few days prior without complaints, and repeat morning ACTH concentration was 35 pg/ml and cortisol concentration was 10.9 μg/dl. Thus, steroid replacement was discontinued. Long-term follow-up of the patient showed that his blood pressure improved and that the patient required only two agents for blood pressure control, chlorthalidone and amlodipine. He no longer required spironolactone or potassium supplements. In addition, he no longer required medications for diabetes and continued to have optimal glucose control.

## Discussion

This case demonstrates the co-secretion of mineralocorticoids and glucocorticoids by benign adrenal adenomas. The patient had a history of severe hypertension and hypokalemia, which was initially attributed to excess aldosterone. Given the large size of the tumor, our index of suspicion for cortisol production was high, and after additional adrenal adenoma workup, subclinical hypercortisolism was also confirmed. Besides hypertension, clinical signs for excess glucocorticoid production included his history of diabetes and obesity.

Co-secreting adrenal tumors have often been described in females, and their common presentation is hypertension (5). Herein, we report on a male patient presenting with hypertension who had a benign co-secreting adrenal adenoma. Most co-secreting adrenal adenomas present as unilateral adenomas (6) (7) (8) (9) (10) (11) (12) (13) (14), but there are also reports of patients with multiple tumors in one adrenal gland (15) (16), bilateral adrenal hyperplasia (17) (18), or single adenomas in both adrenal glands (11) (19). This patient represented a case of a unilateral co-secreting adrenal adenoma.

The etiology for the co-secretion of mineralocorticoids and glucocorticoids has been investigated, and there is evidence that aldosterone-producing adenomas contain more than one cell type besides cells of the zona glomerulosa (5) (20). *In vitro* studies have shown that cells taken from aldosterone-producing adenomas are able to produce cortisol (21). This finding has been confirmed *in vivo* with biochemical evidence of cortisol secretion in a few patients who have presented with primary aldosteronism (8) (12) (21). Multiple studies have used immunostaining as an additional tool to demonstrate the ability of neoplastic cells to produce certain steroids including staining for cholesterol side-chain cleavage (SCC), 3βHSD, CYP17A1, SULT2A1, CYP11B1, and CYP11B2 (11) (12) (13) (14) (15) (16) (17) (19). During histology, the tumor samples obtained from our patient were found to be stained for 3βHSD (Fig. 2A), a precursor to both aldosterone and cortisol. In addition, the tumor displayed positivity for steroidogenic enzymes CYP11B1 and CYP21A2 (Fig. 2B). The immunopositive response of these steroidogenic enzymes confirms the presence of these enzymes, but it cannot necessarily confirm excess hormone secretion.

Co-secreting adrenal adenomas are rarely considered, but need to be recognized when involved in the initial workup for adrenal adenomas. The prevalence of excess glucocorticoids among patients with primary aldosteronism has been reported to be up to 21% (12). This recognition can impact the treatment options in this patient population, especially with postsurgical management. With the removal of a tumor that secretes glucocorticoids, there is concern that patients may develop adrenal insufficiency secondary to the suppression of the hypothalamic–pituitary–adrenal axis. As has been observed in our patient, a low serum cortisol concentration can be observed postoperatively. As noted in the literature, adrenal insufficiency can occur postoperatively in these patients with adrenal tumors co-secreting mineralocorticoids and glucocorticoids (5). Fortunately, there was no evidence of severe adrenal failure following the removal of the tumor in our patient.

## Conclusion

It is important to recognize co-secreting adrenal adenomas. Given that most cases are unilateral, postoperative adrenal crisis or symptomatic adrenal insufficiency can occur in patients who had undiagnosed or untreated adrenal adenomas co-secreting excess mineralocorticoids and glucocorticoids.

## Patient consent

Written informed consent was obtained from the patient for the publication of the case report.

## Author contribution statement

Dr V Yoon and Dr A Heyliger prepared the article. Dr K Carrick, Dr T Maekawa, and Dr H Sasano prepared the adrenal pathology slides. Dr S Woodruff and Dr J Rabaglia carried out the adrenalectomies and Dr R J Auchus and Dr H K Ghayee provided mentorship for writing the article.
